# Female Sex Workers Often Incorrectly Interpret HIV Self-Test Results in Uganda

**DOI:** 10.1097/QAI.0000000000001765

**Published:** 2018-08-14

**Authors:** Katrina F. Ortblad, Daniel K. Musoke, Thomson Ngabirano, Aidah Nakitende, Jessica E. Haberer, Margaret McConnell, Joshua A. Salomon, Till Bärnighausen, Catherine E. Oldenburg

**Affiliations:** * Department of Global Health and Population, Harvard T.H. Chan School of Public Health, Boston, MA; † International Research Consortium, Kampala, Uganda; ‡ Uganda Health Marketing Group, Kampala, Uganda; § Center for Global Health, Massachusetts General Hospital, Boston, MA; ║ Department of Medicine, Stanford University, Stanford, CA; ¶ Heidelberg Institute of Public Health, Heidelberg University, Germany; # Africa Health Research Institute, KwaZulu-Natal, South Africa; ** Francis I. Proctor Foundation, University of California San Francisco, San Francisco, CA; †† Department of Ophthalmology, University of California, San Francisco, San Francisco, CA; ‡‡ Department of Epidemiology & Biostatistics, University of California, San Francisco, CA

***To the Editors:***

## BACKGROUND

HIV testing is important among female sex workers (FSWs) because they are at increased risk of HIV acquisition compared with members of the general population.^1,2^ The World Health Organization recommends that FSWs retest for HIV frequently to detect early HIV infection.^3^ Frequent HIV testing is also important for engagement in HIV prevention interventions, including treatment as prevention^4,5^ and pre-exposure prophylaxis.^6,7^

HIV self-testing is a promising new HIV testing strategy in sub-Saharan Africa (SSA) that has been shown to increase HIV testing in diverse populations.^8–14^ The benefits of HIV testing (eg, initiation of HIV care, prevention behaviors), however, rely on correct interpretation of self-test results. HIV self-testing randomized controlled trials among FSWs in Uganda^13^ and Zambia^14^ found that HIV self-testing achieved near-universal HIV testing coverage and substituted for facility-based testing. In traditional HIV testing and counseling, HIV test results are interpreted by a trained health care professional. With HIV self-testing, the tester must correctly interpret the self-test results without professional assistance and often only the aid of the manufacturer's self-test instructions.

A number of oral HIV self-testing performance studies conducted in SSA found high participant-interpreted HIV self-test sensitivity and specificity (≥94% sensitivity and >98% specificity).^15–24^ In most of these studies, participants received pretest training and interpreted their own self-test result.^15–22,24^ None of these HIV self-testing performance studies were conducted among FSWs,^15–24^ an important key population for HIV prevention interventions.

We explore how well FSWs in Kampala, Uganda, who received pretest training and had 2 previous opportunities to HIV self-test, can interpret images of HIV self-test results.^13^

## METHODS

From October to November 2016, participants were enrolled in a three-armed HIV self-testing cluster randomized controlled trial in Kampala, Uganda.^13^ Eligible participants were: 18 years or older, reported exchanging sex for money or goods (past month), HIV status naive or HIV-negative and did not report recent HIV testing (past 3 months), and Kampala-based.^13^ For this study, we only included participants randomized to the HIV self-testing intervention arms: direct provision of an HIV self-test from a peer educator or provision of coupon exchangeable for an HIV self-test at a health care facility from a peer educator, shortly after enrollment and 3 months later.^13^ The trial used OraQuick Rapid HIV-1/2 Antibody Tests (OraSure Technologies, Bethlehem, PA), which came with a written and pictorial instruction guide (available in both English and Luganda). The trial received ethical approval from Mildmay Uganda and the Harvard T.H. Chan School of Public Health.^13^ All participants provided written informed consent.

We used peer educators to conduct pretest HIV self-test training in a group setting (1 peer educator and 8 participants). The training occurred shortly after enrollment during a peer educator visit that lasted approximately 45 minutes and included information on how to use an HIV self-test and interpret the results. The peer educators had a standardized guide that they were instructed to follow and were observed by research assistants to ensure the quality and consistency of information transmitted.

Participants completed a quantitative assessment at 4 months after enrollment. Here, they were asked to interpret standardized images of HIV self-test results: strong HIV-negative, strong HIV-positive, inconclusive, and weak HIV-positive. Images were presented to scale, in color, on laminated cards and were identical to those included in the manufacturer's instruction guide, which participants received to aid their interpretations. Participants were first shown an image of a strong HIV-positive or strong HIV-negative result. The image presented first reflected the result of their last HIV test, self-reported at 1 month after enrollment. Inconclusive and weak HIV-positive results were next presented in a random order. At 4 months, participants were given the option to complete a rapid HIV test (Alere Determine HIV-1/2, Waltham, MA). We collected electronic data using CommCare (Dimagi, Inc., Cambridge, MA).

We calculated the percentage of participants who incorrectly interpreted each of the self-test results and measured FSW-interpreted HIV self-test sensitivity and specificity. We used participant interpretations of the strong HIV-positive and strong HIV-negative self-test result images to respectively calculate self-test sensitivity and specificity; the interpretation of these images specified in the manufacturer's instruction guide were used as a reference for these measurements. We measured FSW-interpreted HIV self-test negative predictive values and positive predictive values using our sensitivity and specificity measurements and the HIV prevalence of our study population measured at 4 months with rapid HIV testing. Binomial 95% confidence intervals (CIs) were estimated for all measures. We used Stata 13.1 (StataCorp, College Station, TX) for all analyses.

## RESULTS

At enrollment, the majority of participants were younger than 30 years (58%, 314/544), self-reported the ability to read and write (86%, 466/544), completed up to 9 years of education (53%, 286/544), and had previously tested for HIV (95%, 517/544). At 4 months, almost all participants reported using an HIV self-test at least once (95%, 517/544), and participation in rapid HIV testing was 83% (452/544).

**FIGURE 1.**
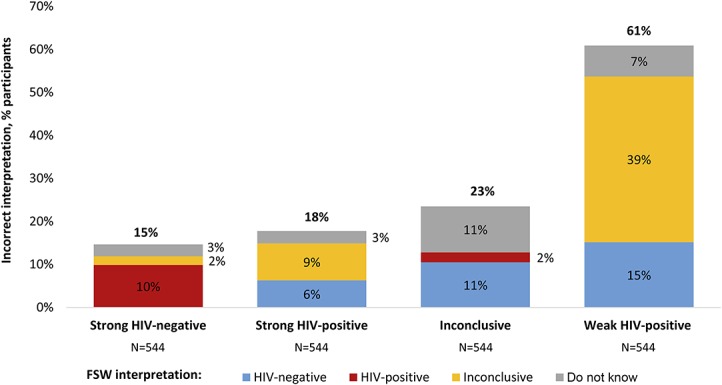
Percentage of FSWs who incorrectly interpreted images of HIV self-test results. The heights of the vertical bars indicate the overall percentage of misinterpreted tests; the color-coded components of the bars indicate the type of misinterpretation: HIV-negative (blue), HIV-positive (red); inconclusive (yellow); do not know (gray).

Figure 1 shows the percentage of participants who incorrectly interpreted the images of HIV self-test results and how each result was misinterpreted. Images of strong HIV-negative, strong HIV-positive, inconclusive, and weak HIV-positive self-test results were incorrectly interpreted by 15% (80/544), 18% (97/544), 23% (126/543), and 61% (328/541) of participants, respectively. The majority of participants (74%, 401/544) incorrectly interpreted at least 1 of the 4 images of HIV self-test results. FSW-interpreted HIV self-test sensitivity was 82% (95% CI: 79% to 85%) and specificity was 85% (95% CI: 82% to 88%), which is also the percentage of participants who correctly interpreted the strong positive and strong negative HIV self-test results, respectively. HIV prevalence among our study participants was 28% at 4 months, which translates into an FSW-interpreted HIV self-test positive predictive value of 68% (95% CI: 64% to 71%) and self-test negative predictive value of 92% (95% CI: 89% to 94%).

## DISCUSSION

Incorrect interpretation of HIV self-test results is common among Kampala-based FSWs, even after pretest training and 2 previous opportunities to HIV self-test. The FSW-interpreted HIV self-test sensitivity and specificity measurements in this study are far below those measured in most of the previous SSA HIV self-testing performance studies.^15–22,24^

Our HIV self-test performance measurements may differ from those in previous studies as a result of differences in pretest training. In previous HIV self-test performance studies, the pretest training provided was often individualized, extensive, and likely unrealistic or too expensive for a scalable HIV self-testing intervention.^15–22^ The peer-led pretest training in this study represents a realistic model for FSWs because peer educators are already a common approach for providing public sector health services to FSWs in SSA.^25–28^ Early at-home pregnancy tests went through a number of redesigns to make the test results more interpretable to users (eg, a plus sign for a positive result; digital results).^29,30^ To reduce misinterpretation of self-test results among FSWs, more research studies should be conducted on the design of HIV self-tests, the appropriate level of pre-test training, and the usefulness of on-demand support.

Methodological differences between our study and previous HIV self-testing performance studies may additionally explain our lower HIV self-test performance measurements. In our study, participants interpreted images of HIV self-test results rather than self-tests used to test themselves. In previous studies, measurements of self-test performance may have been biased because participants' previous knowledge of their HIV status may have influenced their interpretation of self-test results.^15–24^ Understanding how well individuals can interpret HIV self-test results without the influence of previous HIV status knowledge is important because HIV self-testing has the potential to move HIV testing outside the health care system.^13^ In this unregulated environment, individuals may use HIV self-tests for first-time HIV testing or to test the HIV status of other individuals, such as a child or sexual partner.

Unique characteristics of FSWs may also explain the lower HIV self-test performance measurements in this study. Compared with other populations, FSWs may have challenges interpreting HIV self-test results for reasons including lower levels of health literacy,^31^ higher prevalence of substance use,^32–34^ and differences in educational attainment.^35–37^

Concerns related to incorrect interpretation of HIV self-test results vary, based on which results are misinterpreted and how they are misinterpreted. Participant misinterpretation of inconclusive and weak HIV-positive self-test results was common, but in real-world settings, these results are rare.^16,17,20,22^ Participant misinterpretation of strong HIV-negative and strong HIV-positive self-test results was less common, but more concerning: false perceptions of HIV-positive status may cause emotional distress,^38^ result in stigma and discrimination,^39^ and alter prevention behaviors,^40–43^ whereas false perceptions of HIV-negative status may delay linkage to care, increasing the risk of poor health outcomes^44^ and secondary transmission of HIV.

This study has several limitations. First, participants did not interpret self-test results in a random order and thus, exposure to previous results may have influenced interpretations of later results.^45^ Second, we did not collect self-tests used by participants and thus were unable to measure the prevalence of weak HIV-positive and inconclusive self-test results. Third, participants may have paid less careful attention when interpreting an image of a self-test result rather than their own self-test result.

HIV self-testing has the potential to dramatically increase HIV testing and aid in the achievement of 90% HIV status knowledge among all individuals living with HIV by 2020.^46^ The effect of HIV self-testing may be diminished, however, if self-testers do not correctly interpret self-test results. To avoid misinterpretation of HIV self-test results that can result in false perceptions of HIV status, policy makers should considering implementation of realistic pretest training and on-demand HIV self-test support, whereas HIV self-test manufacturers consider redesign of HIV self-tests.
